# Virus-Specific T-Cell Therapy for Viral Infections of the Central Nervous System: A Review

**DOI:** 10.3390/v15071510

**Published:** 2023-07-06

**Authors:** Nicolas Lambert, Majdouline El Moussaoui, Frédéric Baron, Pierre Maquet, Gilles Darcis

**Affiliations:** 1 Department of Neurology, University Hospital of Liège, 4000 Liège, Belgium; pmaquet@uliege.be; 2 Department of General Internal Medicine and Infectious Diseases, University Hospital of Liège, 4000 Liège, Belgium; melmoussaoui@chuliege.be (M.E.M.); gdarcis@chuliege.be (G.D.); 3 Department of Hematology, University Hospital of Liège, 4000 Liège, Belgium; f.baron@chuliege.be

**Keywords:** virus-specific T-cells, T-cell transfer, immune therapy, cellular therapy, viral infections, central nervous system, progressive multifocal leukoencephalopathy, cytomegalovirus, adenovirus, encephalitis, multiple sclerosis

## Abstract

Opportunistic viral infections of the central nervous system represent a significant cause of morbidity and mortality among an increasing number of immunocompromised patients. Since antiviral treatments are usually poorly effective, the prognosis generally relies on the ability to achieve timely immune reconstitution. Hence, strategies aimed at reinvigorating antiviral immune activity have recently emerged. Among these, virus-specific T-cells are increasingly perceived as a principled and valuable tool to treat opportunistic viral infections. Here we briefly discuss how to develop and select virus-specific T-cells, then review their main indications in central nervous system infections, including progressive multifocal leukoencephalopathy, CMV infection, and adenovirus infection. We also discuss their potential interest in the treatment of progressive multiple sclerosis, or EBV-associated central nervous system inflammatory disease. We finish with the key future milestones of this promising treatment strategy.

## 1. Introduction

With the rising number of patients being under immunosuppressive treatments for autoimmune diseases or organ transplantation as well as with the growing life expectancy of individuals with hematological malignancies and solid cancers, opportunistic infections of the central nervous system (CNS) cause significant morbidity and mortality [1,2]. Indeed, even in high-income countries, these CNS opportunistic infections continue to carry a particularly poor prognosis, as highlighted by a recent French study published in 2023 reporting a one-year mortality rate of 38.2% for patients diagnosed with progressive multifocal leukoencephalopathy (PML) between 2008 and 2017 [3]. CNS infections in immunocompromised patients raise several diagnostic challenges, including the possible lack of typical infectious features such as fever or increased white blood cell count in the cerebrospinal fluid (CSF) in individuals with cytopenia, the frequent multiple concurrent infections, the virulence of usually harmless pathogens, and the multitude of possible differential diagnoses, including drug toxicities [1,2]. Moreover, the treatment of CNS opportunistic viral infections is challenging because no antiviral treatment has been proven effective in clinical settings, apart from human immunodeficiency virus (HIV) and some herpesviruses [4]. Hence, the sustained control of opportunistic CNS viral infections typically relies on the ability to restore adequate antiviral immunity [3,5]. In this context, new treatment paradigms which aimed at restoring this antiviral immune activity have been developed, including adoptive virus-specific T-cell transfer. In contrast to old beliefs that CNS immune privileged status prevented it from T-cell entrance, it is now established that T-cells can enter and traffic into the CNS, either through the choroid plexus or through the blood–brain barrier and the glia limitans, both in basal and inflammatory conditions. This trafficking requires the sequential occurrence of only partially known tightly regulated processes (reviewed earlier in [6,7,8]). Therefore, T-cell trafficking into the CNS should not represent a serious limitation to this treatment strategy. In this manuscript, we first describe the methods used to develop or select virus-specific T-cells and then review the clinical evidence for their use in CNS infections.

## 2. Development and Selection of Virus-Specific T-Cells

**Figure 1:**
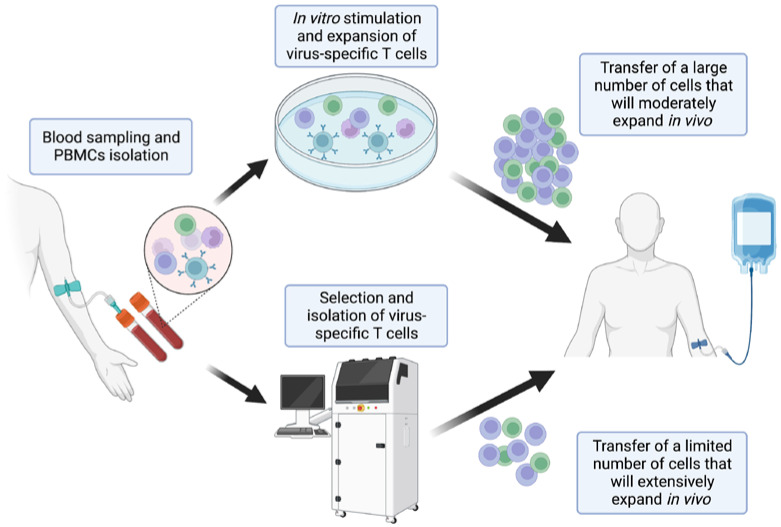
Methods used to produce virus-specific T-cells. First, blood is collected from the T-cell donor, which can be either the patient themself, the transplant donor in the case of hematopoietic stem cell donor, or a third party. Peripheral blood mononuclear cells (PBMCs) are isolated. These can be cultured in vitro for several days to weeks in the presence of virus antigens to produce large amounts of cells that will be infused to the patient but will usually expand only moderately in vivo. Another approach is the direct selection of virus-specific T-cells from the collected PBMCs. Several methods exist for this process, the most widely used being the cytokine-capture method, described in the text. After selection of a limited number of specific T-cells, these are infused to the patient where they can expand extensively.

The first clinical experience of T-cell transfer for the treatment of an opportunistic infection (more precisely in this case, of a virus-induced neoplasm) was reported in the 1990s when donor lymphocyte infusions (DLIs) were used in allogeneic hematopoietic stem cell transplantation (HSCT) recipients to treat EBV-associated post-transplant lymphoproliferative disorders (PTLDs) [9]. Although promising, DLIs were not specific to any epitope and, therefore, were associated with a significant risk of graft-versus-host disease (GvHD) occurrence [10]. Since then, two main paradigms have been developed to isolate or enrich virus-specific T-cells: the expansion of virus-specific cells in vitro before infusion and the selection of virus-specific cells that will expand in vivo after infusion (Figure 1) [11].

In vitro stimulation and expansion was the first reported method to obtain virus-specific T-cells in order to prevent cytomegalovirus (CMV) infection in three HSCT recipients [9]. Donor peripheral blood mononuclear cells (PBMCs) were cultured in the presence of CMV-infected autologous fibroblasts, then clonally expanded, and finally selected to obtain CMV-specific CD8+ T-cells [12,13]. All three patients showed evidence of reconstitution of CMV-targeted CD8 T-cell activity and none of them presented any significant adverse event [12]. However, the presence of virus-specific CD4+ T-cells seemed to be needed for the persistence of the transferred CD8+ T-cells and prolonged infection control [8]. Hence, protocols aiming at producing polyclonal CD4+ and CD8+ CMV-specific T-cells were established, as well as protocols using antigen-presenting cells pulsed with viral antigens in order to free the production system from live virions [14,15]. 

As in vitro clonal expansion of cultured T-cells was associated with an increased expression of Fas, resulting in more rapid cell death, and decreased expression of CD28, probably resulting in a reduced activity, protocols to directly select virus-specific T-cells from seropositive donors, that are thought to subsequently proliferate more extensively in vivo, have emerged. Although different methods to select T-cells exist, the most widely used approach is the cytokine-capture method [11,16,17]. Donor PBMCs are stimulated with antigens specific to the virus of interest. Anti-CD45 (expressed on leukocytes) antibodies conjugated with anti-gamma interferon antibodies are used to attach gamma-interferon-producing T-cells. Virus-specific T-cells (those that produce greater amounts of gamma-interferon after antigenic stimulation), are then labelled using anti-gamma interferon magnetic antibodies and then sorted with magnetic columns. Hence, specific T-cells are obtained in limited proportions and are infused to the recipient where they can expand and, hopefully, durably control the infection.

In addition to CMV, various similar protocols have been published to produce T-cells specific to several other viruses including Epstein–Barr virus (EBV); human polyomavirus 1 (HPyV1, also known as BK virus) and 2 (HPyV2, also known as JC virus); human adenoviruses; human herpesvirus 6 (HHV6); hepatitis B virus (HBV); HIV; and severe acute respiratory syndrome coronavirus 2 (SARS-CoV-2) [18,19,20,21,22,23]. It is worth noting that although not reviewed in this manuscript, recent techniques which aim at producing multi-virus-specific cells have also been developed [20,24,25,26]. Finally, another recent important advancement is the development of virus-specific T-cells from third-party donors, which not only offers the opportunity to propose this therapeutic approach to patients other than allogeneic HSCT recipients, but also opens the possibility to set up banks of timely available specific T-cells [20].

## 3. Clinical Experience in CNS Infections

### 3.1. Progressive Multifocal Leukoencephalopathy

Progressive multifocal leukoencephalopathy is a rare but often fatal brain infectious disease caused by HPyV2 in immunocompromised individuals. HPyV2 establishes lifelong latent infection in most of the general population but, in the course of cellular immune deficiency, it can reactivate, replicate, undergo complex genetic rearrangements, and, in that way, acquire the ability to cause lytic infection of the oligodendrocytes [27]. Nowadays, acquired immunodeficiency syndrome, hematological malignancies, chronic inflammatory diseases under immunosuppressive therapy (notably natalizumab for multiple sclerosis), solid organ transplantation (SOT), solid neoplasms, and primary immune deficiencies account for the vast majority of PML cases [3]. As there is no antiviral treatment proven effective for PML, the prognosis mainly relies on the ability to achieve timely immune reconstitution. In this line, several strategies aimed at reinvigorating anti-HPyV2 immune activity have recently emerged, including the use of immune checkpoint inhibitors, human recombinant interleukin 7, and polyomavirus-specific T-cell transfer [28,29,30,31,32,33].

In 2011, for the first time, Balduzzi and colleagues reported the clinical course of a 19-year-old HSCT recipient who developed PML after several years of immunosuppressive therapy for GvHD and who was successfully treated with HPyV2-specific T-cells [34]. These cells were obtained in vitro by culturing PBMCs from the bone marrow donor with peptide pools spanning the entire VP1 and large tumor proteins, which are two immunogenic proteins expressed on the surface of HPyV2. Specific T-cells were successfully produced, and were able to proliferate, produce gamma interferon, and specifically exert cytotoxicity in response to HPyV2 antigenic stimulation. They were infused to the patient, which reportedly resulted in a remarkable neurological clinical improvement, a drastic decrease in CSF HPyV2 load which was no longer detectable, and a halt in lesions’ progression visualized by MRI, which naturally evolved into brain atrophy. Lymphocytes collected from the patient after T-cell infusion also showed activity against HPyV2, which was not present before infusion, and the patient also showed a high intrathecal HPyV2-specific antibody production, mirroring humoral immune reactivity. No GvHD or other adverse event was reported. Although these results were promising, it should be noted that the patient was treated with cidofovir, an agent showing antiviral activity in vitro against HPyV2, and that immunosuppressive therapy had recently been stopped, which could have participated in the antiviral immune reinvigoration as well as in the favorable clinical course of the patient. However, both cidofovir initiation and immunosuppressive therapy withdrawal occurred 2 months before T-cell infusion, with still no neurological improvement and no specific antiviral immune activity detectable at the time of T-cell therapy onset. The authors, therefore, postulated that the patient’s recovery was, at least partly, due to the cell therapy.

In 2018, Muftuoglu and colleagues published a series of three patients who received cryopreserved third-party HPyV1-specific T-cells to treat PML [35]. HPyV 1 and 2 are genetically similar and show a high degree of sequence homology for several immunogenic proteins such as the large tumor antigen or VP1 [36,37,38,39]. As a result, HPyV 1- and 2-specific T-cells show cross-reactivity. Among the three reported patients, two had hematological malignancies (acute myeloblastic leukemia and polycythemia vera) and one was infected with HIV and had stopped antiretroviral therapy five years earlier. Two patients showed clinical improvement following T-cell administration, and the last one showed stabilization of the previously progressing disease. The HIV-infected patient showed a slight clinical worsening before improvement, and contrast-enhancement after gadolinium injection was seen on MRI, two findings evoking immune reconstitution inflammatory syndrome (IRIS). No other relevant adverse event was reported. The HLA-Bw mismatch between one patient and the T-cell donor allowed the authors to study T-cell persistence after infusion as well as their trafficking into the CSF. It was shown that infused T-cells rose to almost 300 times their original number by day 14 after infusion and that virus-specific cells successfully trafficked into the CSF [35].

**Figure 2:**
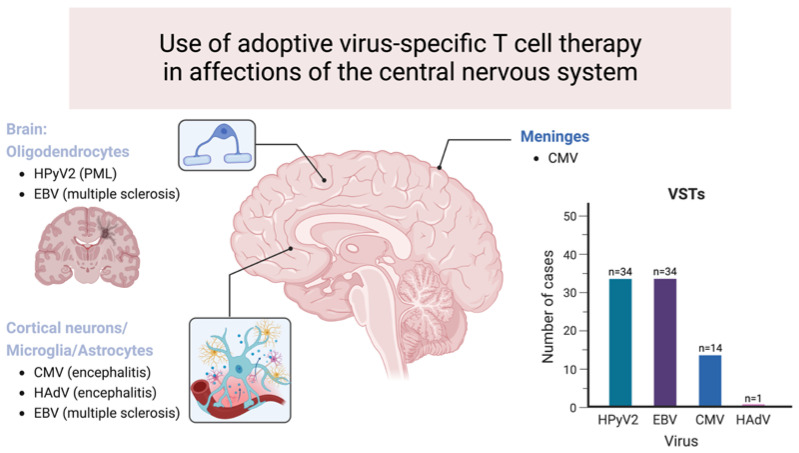
Use of adoptive virus-specific T-cell therapy in affections of the central nervous system. Central nervous system affections for which the use of virus-specific T-cell therapy has been reported are represented in this figure. The graph on the right shows the number of cases reported per virus. HAdV = adenovirus; CMV = cytomegalovirus; EBV = Epstein–Barr virus; HPyV2 = Human polyomavirus 2; PML = progressive multifocal leukoencephalopathy; VSTs = virus-specific T-cells.

To date, at least 34 patients with PML treated using human polyomavirus (either HPyV1 or HPyV2) -specific T-cells were reported in four case reports and five case series (Table 1, Figure 2) [34,35,40,41,42,43,44,45,46]. Among them, 22 patients received HPyV1-specific T-cells while the 12 others received cells targeting HPyV2. Four patients received autologous T-cells, three received allogeneic T-cells from their HSCT donor, and 27 patients received third-party allogeneic T-cells. The cause of immune deficiency was an hematological malignancy for 23 patients (67,5%; among which 26% had been treated with allogeneic HSCT and 30% with autologous HSCT), primary immunodeficiency for five patients (14.7%), autoimmune disease for three patients (8.8%, including one patient with both autoimmune disease and solid cancer), HIV infection for one patient (3%), SOT for one patient (3%), and chronic HBV and HDV infection for one patient (3%).

Among the 34 patients, 21 showed a favorable clinical course following T-cell administration: 16 of them showed neurological improvement and 5 stabilized a previously progressing disease. Eleven patients continued to deteriorate despite cellular therapy and died of PML. The outcome regarding PML is impossible to specify in two patients: one patient was lost to follow-up and the other died of Varicella–Zoster virus infection shortly after the first infusion [40,41]. Two patients presented IRIS: death was at least partially imputed to IRIS for one patient [35,45]. No other clinically relevant adverse event was noted, including no GvHD or autoimmune disease flare.

It is worth noting that the average time between PML diagnosis and first T-cell infusion was 2.6 months, resulting in a potential selection bias excluding the most impaired patients. Hence, in the larger series reported so far, among 26 patients initially assessed for eligibility, 6 died before treatment initiation [40]. This treatment delay may also prevent patients from useless treatments, as three patients stabilized spontaneously without any treatment. However, it also implies that cell therapy is probably not the most suited for rapidly progressive diseases. To overcome this obstacle, one option could be to create banks of ‘ready for use’ frozen third-party specific T-cells. However, this therapeutic strategy has not yet been implemented enough in routine clinical practice to establish these banks. Moreover, a standardization in cell production and administration is required. Nonetheless, although several challenges still need to be overcome for large-scale optimal use of this treatment, T-cell transfer for PML seems to be a promising strategy that should be further investigated in randomized clinical trials.

### 3.2. Human Adenovirus Infection

Human adenovirus (HAdV) infections are common in the general pediatric population and more than 80% of children aged 1 to 5 years show neutralizing antibodies to one or more HAdV serotypes [47]. Unlike other DNA viruses, HAdV genome double-stranded DNA is not integrated into the host cell DNA but remains in an episomal state allowing life-long persistence, mainly in T-lymphocytes [48]. Among immunocompetent hosts, infection is often asymptomatic or mildly symptomatic but, in the setting of a reduction of specific T-cell immunity, the virus may reactivate, replicate, and cause HAdV disease [49]. The latter represents a significant cause of morbidity and mortality in immunocompromised pediatric patients, especially between two and three months following allogeneic HSCT, occurring in 15–23% of these situations [50,51]. In the absence of effective T-cell immunity, infection can result in a broad clinical spectrum ranging from asymptomatic viremia to invasive disease including hemorrhagic enteritis or cystitis, hepatitis, nephritis, pneumonia, myocarditis, and CNS infections, with a mortality rate that may reach 26% [52,53]. HAdV-related CNS infection can manifest itself from self-limited febrile seizures, aseptic meningitis, or reversible encephalitis to devastating acute necrotizing encephalopathy that may be fatal within a few days [54,55,56,57]. The main risk factors for the development of HAdV infection include the use of T-cell–depleted grafts for allogeneic HSCT, the type of donor (unrelated donor, cord blood, haploidentical or HLA mismatched parent-child), and profound lymphopenia in the first months post-HSCT [58].

In these patients, it is crucial to identify early HAdV infection and adopt measures to reduce viral replication, preventing it from becoming disseminated and causing life-threatening disease. As in other viral infections in HSCT settings, the possible treatment paradigms include prophylaxis, preemptive treatment, and therapy of already established HAdV disease. Therapeutic strategies include tapering of immune suppression, use of antivirals, and promoting immune recovery with adoptive immunotherapy. Although there are currently no specific antivirals approved for HAdV infections, cidofovir is the most used agent, but is associated with a significant nephrotoxicity and poor response rates in patients awaiting immune reconstitution of the T-cell compartment [59,60]. Moreover, this drug is poorly investigated in the treatment of CNS infections, and pharmacological data on CSF or brain penetration are lacking [61]. Brincidofovir, an oral lipid-conjugated prodrug of cidofovir, has superior bioavailability and CSF penetration compared to cidofovir, but its use is currently limited by its poor availability in several countries [60,62,63]. Hence, as antiviral treatments appear of limited efficacy, clearance of HAdV infection in HSCT recipients relies mainly on the ability to achieve cellular immune reconstitution. In the past few years, allogeneic virus-specific T-cell transfer has appeared as a safe and effective treatment for double-stranded DNA virus infections in immunocompromised patients with minimal risk of developing GvHD.

As described above, adoptive transfer of HAdV immunity in the allogeneic HSCT setting was first based on unselected DLI, but the potentially high frequency of alloreactive T-cells and the ensuing side effects are major impediments to this approach [64,65,66,67]. The isolation of HAdV-specific T-cells from peripheral blood of the original stem cell donor has, therefore, become the method of choice [59]. One of the early successful attempts at adenovirus-reactive T-cell isolation was described by Feuchtinger and colleagues using the interferon-γ (IFN-γ) capture system (Miltenyi Biotec, Bergisch Gladbach, Germany) after short (range 7–29 days with a median of 18 days) in vitro stimulation with HAdV antigens [68,69]. Using this method, even low numbers of transferred HAdV-specific T-cells could expand in vivo, and HAdV-specific T-cell immunity could be detected for up to 6 months, indicating a sustained and long-term protective T-cell response. The most critical parameter for treatment success was the early onset of adoptive T-cell transfer, when the immune system had time to limit the infection and subsequent organ damage [69]. A similar approach of enrichment of IFNγ-secreting cells but with shorter ex vivo expansion (HAdV-specific T-cells were infused directly after 16 h of stimulation) was evaluated in 30 HSCT recipients with HAdV disease or viremia [70]. In vivo, expansion of HAdV-specific T-cells was obtained in 61% of patients and resulted in complete clearance of viraemia in 86% who achieved T-cell expansion, with no reported acute toxicities or significant onset of GvHD. More recently, faster manufacturing protocols and third-party banks of ready-to-use specific T-cells have been developed to make this treatment more accessible (although still unavailable at most centers). Many recent studies of both donor-derived and third-party virus-specific T-cells suggest that specific T-cells are safe and effective for the treatment of HAdV infection in immunocompromised hosts with reported response rates ranging from 70 to 91% [71,72,73,74]. These data suggest that HAdV-specific T-cell therapy is a promising approach, especially as a preemptive treatment in the case of viraemia [75]. Certain algorithms already suggest their use in case of HAdV infection or viraemia not responding to antiviral agents in patients lacking circulating HAdV-specific T-cells [49]. However, few data are available concerning CNS-related infections [70,76]. Among the 30 patients reported here above, only one had encephalitis and died two days after treatment administration, before the latter could have been beneficial, preventing us from drawing any conclusion on the safety and efficacity of this approach (Figure 2) [70]. It is reasonable to postulate that T-cell therapy might mostly benefit patients with mild to moderate forms of CNS infections, as acute necrotizing encephalopathy would usually be fatal before cell administration or expansion. Further studies are, therefore, needed to investigate the use of virus-specific T-cells for CNS HAdV infection.

### 3.3. Cytomegalovirus Infection

CMV infection is a significant cause of morbidity and mortality in patients with severe immune suppression. Solid organ transplantation, HSCT, and acquired immunodeficiency syndrome are responsible for the majority of CMV infections. CMV reactivation occurs in up to 70% of seropositive HSCT recipients in the absence of antiviral prophylaxis and in approximately 40% of HIV-infected patients with advanced immunosuppression before the introduction of ART [77,78,79,80]. Risk factors associated with CMV reactivation in HSCT setting include seropositivity of the recipient (particularly in the case of a seronegative donor), cord-blood transplant, haploidentical HSCT, an HLA-mismatch unrelated donor, and use of high-dose corticosteroids, anti-thymocytes globulin, mycophenolate mofetil, or post-transplant cyclophosphamide [81,82]. For solid organ transplant recipients, seropositivity of the donor and lung and small intestine transplants are associated with the highest risk of CMV reactivation, while heart and kidney recipients have a lower risk [83]. The digestive tract and lungs are the most commonly involved organs during CMV infection after HSCT, while CNS disease is rare [84,85,86]. CNS involvement includes mainly isolated encephalitis, but can also present as meningitis, ventriculitis, cerebral mass lesions, polyradiculomyelitis, and transverse myelitis [85]. CMV encephalitis is a relatively late complication after HSCT, usually occurring over four months after transplantation [84].

So far, there have been no controlled, randomized, double-blinded studies investigating the safety and efficacy of antiviral agents in patients with CMV infection. Current management focuses on both the reduction of immunosuppressive therapy and the initiation of antiviral therapies including intravenous ganciclovir, foscarnet, or maribavir [85,86,87,88,89]. Letermovir, recently FDA approved for CMV prophylaxis following HSCT, has not been approved yet for the treatment of drug-resistant CMV [90]. Despite recent advances in pharmacologic therapies, CMV encephalitis continues to be associated with high morbidity and mortality rates, notably because of CMV-specific immunity impairment, drug toxicity, selection of resistance mutations, and poor drug bioavailability into the CSF [84]. Although the overall incidence of drug-resistant CMV remains low (0–8%), it reaches 14.5% in high-risk populations such as haploidentical HSCT receiving antiviral prophylaxis [91,92]. Consequently, adoptive cellular therapy appears as a promising and increasingly available treatment of CMV infections refractory to standard antiviral therapy.

The use of CMV-specific T-cells in HSCT recipients has been well documented, evolving from initial case reports to Phase I and II trials [93]. The use of specific T-cells as antiviral prophylaxis after HSCT has first been explored by Riddell and colleagues by infusing CMV-specific T-cell clones derived from bone marrow donors, but this approach was not applied on a larger scale because of the technical challenges associated with cell cloning [12]. One of the first reports describing donor-derived CMV-specific T-cells generated by ex vivo expansion as therapy included eight adult HSCT recipients with refractory or resistant CMV infection. Six patients (75%) demonstrated virologic clearance after treatment, with no evidence of de novo GvHD [14]. Since then, many reports including more than 450 HSCT recipients with CMV infection or disease have described the efficacy of CMV-specific T-cells in clearing active infection with response rates ranging between 70% and 90% [94,95,96,97,98].

Several cases of CMV-related neurological infections treated with specific T-cells have been reported in the literature (Figure 2). Feuchtinger and colleagues reported 18 HSCT recipients with refractory CMV infection or disease treated with T-cells produced on a short-term trial thanks to a protocol using IFN-γ secretion of T-cells after ex vivo stimulation with viral A [94]. Among these 18 patients, two had CMV encephalitis and cleared the infection in four weeks after adoptive T-cell transfer. The cells originated from a third-party donor for one patient and from the stem cell donor for the other one. Unfortunately, one of them died of renal failure 425 days after HSCT, without further information available. Prockop and colleagues also reported eight cases of drug-refractory CMV meningoencephalitis treated with adoptive transfer of banked third-party using CMV pp65-sensitized T-cells [99]. Six ultimately obtained viral clearance and are long-term survivors (two years) after treatment. Two other cases of pediatric patients successfully treated with third-party CMV-specific T-cells for drug-refractory CMV meningoencephalitis have been reported [100]. Interestingly, in one of them, persistence of the third-party donor lymphocytes population was demonstrated in blood for at least 7 months post-infusion and the patient did not experience any further significant CMV reactivation during this time period. Finally, Ke and colleagues reported two patients with drug-resistant CMV encephalitis after HSCT successfully treated with donor CMV-specific cytotoxic T-lymphocytes in combination with antiviral drugs [101]. Assuming that CMV-specific cytotoxic T-cells might potentially induce or aggravate GvHD, the authors proposed intrathecal administration of CMV-specific T-cells in combination with intravenous anti-viral therapy for one of the patients who had Grade 3 GvHD [102,103]. Administration of CMV-specific cytotoxic T-cells was followed by the decrease of CMV viral load in CSF while not aggravating GvHD symptomatology. Therefore, the authors concluded that intrathecal administration of CMV-specific T-cells may represent a therapeutic option, especially for patients with severe GvHD. This type of administration could also protect the infused cells from the deleterious effects of the ongoing immunosuppressive therapies [103]. However, the safety and efficacy of intrathecal CMV-specific T-cell administration need to be confirmed by further studies. Several questions remain regarding the optimal dosing regimens, route of administration, timing of administration, potential benefits of combining T-cells with anti-viral therapy, and appropriate cell generation methodology. Nevertheless, despite these limitations, virus-specific T-cells have shown promising results for both prophylactic use and for the treatment of CMV infection, particularly in the setting of refractory or resistant diseases.

## 4. EBV-Specific T-Cell Therapy for Multiple Sclerosis

Multiple sclerosis (MS) is the most prevalent CNS chronic inflammatory disease, affecting around 2.8 million people worldwide [104]. It is characterized by areas of inflammatory demyelinating lesions and axonal transections, called “plaques”, within both white and grey matter. The clinical course of MS is highly variable but can be roughly divided in a relapsing–remitting pattern and a progressive pattern, although these phenotypes can largely overlap in clinical practice. MS typically presents in young adults, especially women, and leads to lifelong disability and cognitive impairment that affect both quality of life and life expectancy [105]. Several classes of disease-modifying therapies are available and effective for the treatment of MS, essentially for its relapsing–remitting form. While some of these treatments seem to be partially effective for early progressive MS, especially those with prominent inflammatory features, no highly effective treatment exists for the progressive form of MS.

The etiology of MS is far from fully understood and probably complex and multifactorial, implicating both genetic and environmental factors. Pierre Marie suggested an infectious etiology for MS in 1894 and since then, a myriad of pathogens have been proposed to be implicated in its pathophysiology [106]. Among these, EBV is probably the pathogen with most epidemiological, serological, and virological evidence supporting its role in MS. EBV, or human herpesvirus 4, is a ubiquitous gamma herpesvirus infecting over 90% of the adult population worldwide and is associated with a wide range of cancers including notably nasopharyngeal carcinomas, Hodgkin and non-Hodgkin B lymphomas, certain gastric carcinomas, NK/T-cell lymphomas, and leiomyosarcomas [106,107,108]. Following lytic primary infection of the oral mucosal epithelium, either asymptomatic in children or causing infectious mononucleosis in young adults, EBV establishes long-term latent infection by inducing lifelong transformation of infected B-cells. Hence, EBV reprograms naïve B-cells towards a developmental path comprising a proliferative state, a germinal center-like phenotype, and a memory B-cell phenotype. These developmental stages correspond to different viral gene programs called “latency profiles” [106,109]. The proliferative stage shows a so-called latency III profile, characterized by the expression of the proteins LMP1 and 2 and EBV nuclear antigens (EBNA) 1 to 6. The germinal center phenotype shows a latency II profile showing expression of LMP1 and 2 and EBNA1. Finally, the memory phenotype shows a latency I/0 profile with a gene expression limited to EBNA1. Interestingly, these latency profiles also correspond to distinct EBV-associated malignancies. While the latency III profile is found in lymphoproliferative diseases arising in immunocompromised individuals, most notably PTLDs, latency II and I profiles are found in EBV-associated cancers of immunocompetent patients [109,110,111,112]. EBNA 3, 4, and 6 are highly immunogenic which can explain that cancers associated with a latency III profile almost only develop in immunocompromised individuals [113,114]. Conversely, LMP1 and 2 and EBNA1 show various effective mechanisms of immune evasion, limiting the elimination of cells with latency II or I profile, especially in genetically-susceptible individuals [106]. However, it was shown that in vitro expansion and maturation of specific T-cells can overcome these immune evasion mechanisms [115].

In a recent cohort study of 10 million US army personnel longitudinally followed for more than 20 years, it was shown that EBV infection is a precondition for MS development and that seroconversion in young adulthood increases the risk of MS by 32-fold [116]. Moreover, although challenged by other reports, the expression of EBV proteins including LMP1, LMP2A, EBNA1, and EBNA2 was detected in MS brain lesions with co-localization of autoreactive T-cells [117,118,119,120,121,122]. Still, the precise link connecting EBV and MS is poorly understood. Several hypotheses exist, including molecular mimicry, immortalization, and rescue of autoreactive B-cells or deregulation of B-cell gene expression coding for immune control (reviewed earlier in [106]). Among these hypotheses, the role of a deficient immune control of EBV-infected cells harboring latency II/I phenotypes has repeatedly been mentioned [123,124]. Accordingly, strategies aimed at enhancing EBNA1 and LMP1 and 2 T-cell immunity have been considered. Since EBV-associated PTLDs are already being treated with EBV-specific T-cell transfer, it was postulated that similar techniques could be used to treat MS and especially its progressive form, which has few treatment options. In order to produce EBNA1 and LMP1- and 2-specific T-cells, the AdE1-LMPpoly vector was designed, encoding a modified EBNA1 gene limiting immune evasion fused to a polypeptide of 16 MHC class I-restricted epitopes derived from LMP 1 and 2A [125]. This vector was proven very efficient for the expansion of EBNA1 and LMP1 and 2-specific T-cells [126,127,128].

Using this technology for the first time in 2013, a patient with secondary progressive MS was treated using autologous EBV-specific T-cells (Figure 2) [129]. Before treatment, the patient was unable to walk or transfer himself, had trigeminal neuralgia, intension tremor limiting the use of his hands and urinary incontinence requiring a permanent urinary catheter. His Expanded Disability Status Scale (EDSS) score was 8.0. The proportion of his blood EBV-specific CD8+ T-cells was under the 10th percentile for healthy EBV carriers. After blood collection, EBV-specific T-cells were stimulated and expanded in vitro using AdE1-LMPpoly and interleukin-2. After expansion, 38.46% of the CD8+ T-cells but only 0.22% of CD4+ T-cells were reactive to LMP peptides. The patient received four escalating doses (from 5 × 10^6^ to 2 × 10^7^ cells) infused every two weeks. Following the treatment, he experienced a reduction in his fatigue and lower limb spasms, improvement in cognition, increased productivity at work, and improvement in hand functions and lower limbs voluntary movements. The frequency of blood EBV-specific CD8+ T-cells also increased, the number and size of gadolinium-enhancing brain lesions visualized with MRI decreased, as well as the intrathecal IgG production. These observations were sustained for 3.5 years after treatment completion. Stimulated by this first experience, an open-label Phase I study was set up, using four escalating doses of autologous EBV-specific T-cells to treat ten patients with progressive MS (five with a primary progressive phenotype and five with a secondary progressive phenotype) [127]. Of these ten patients, seven reported a subjective improvement and six demonstrated objective clinical improvement including three who improved in the EDSS score. Of the three patients not showing improvement, two remained clinically stable and only one patient experienced clinical deterioration. Fatigue was the most prominent ameliorated feature and clinical improvement was associated with a positive progress of the quality of life. In addition, three patients showed reduction of the IgG intrathecal production. Follow-up analyses showed that at least some degree of clinical improvement was still observed in four participants after two years and in three participants after three years [130]. The clinical response was dependent upon the quality of the infused product. Hence, all six patients receiving T-cells with strong EBV reactivity (>5% of CD8+ T-cells) showed clinical improvement in comparison with only one patient receiving T-cells with weak EBV reactivity [127]. No serious treatment-related adverse event was reported during the three-years follow-up.

Important limitations were the inability to generate EBV-specific T-cells from two patients, likely due to the lack of HLA matching of the patients with T-cell epitopes encoded in the vector, as well as the relatively low EBV specificity of the final product in certain participants. To overcome these difficulties, a product of allogeneic off-the-shelf EBV-specific T-cells produced using AdE-LMPpoly vector, called ATA188, was developed, which allowed selecting optimal donors and offered a practical, rapid, and widespread use across autologous therapies. A Phase I open-label study assessing its safety in 24 patients with progressive MS has been completed and found no treatment-related serious (>grade 3) adverse event [131]. In total, 9 of the 24 treated patients showed sustained disability improvement, mirrored either by a decrease in the EDSS score or by a reduction in the time needed to walk 25 feet. Thirteen patients showed stable EDSS scores and only four patients experienced progression. Five patients maintained their clinical improvement after 4 years of follow-up.

## 5. Safety

Adoptive immunotherapy using virus-specific T-cells has been explored for over 2 decades and, until now, it has shown remarkable safety for the treatment of virus-associated diseases [132,133,134,135,136,137]. Few mild-to-moderate adverse events were reported, including GvHD and infusion toxicity for most cases, generally showing a favorable outcome with standard-of-care treatment [132]. Although GvHD has been a primary concern for the use of adoptively transferred allogeneic T-cells, especially in the third-party setting, the rates of reported GvHD in clinical trials have been low [132]. This can be explained by a reduced diversity of T-cell receptor (TCR) repertoire used to generate virus-specific T-cells leading to a lower potential for alloreactivity compared to the CD3-activated T-cells generally used to produce chimeric antigenic receptor (CAR) T-cells [138,139]. Although several studies reported cases of grade I to III GvHD after virus-specific T-cells therapy in HSCT recipients [20,70,71,74,140], most of these reports lacked control groups, making it difficult to determine whether GvHD arose from virus-specific T-cell infusion or from the HSCT itself [70], especially since most patients who developed GvHD had prior risk factors for its occurrence [114,141]. While some studies have shown in vitro cross-reactivity of virus-specific T-cells with recipient antigens, it was correlated with an increased risk of GvHD in vivo [142,143]. Still, because of this concern for alloreactivity, most studies on virus-specific T-cells excluded patients with active grade III or IV GvHD. Hence, further studies are needed to determine the safety of virus-specific T-cell therapy in the setting of severe GvHD. Concerning infusion toxicities, very few cases of isolated fever were identified [20,132,144,145]. No evidence of cytokine release syndrome (CRS), a complex hyperimmune response that may occur after receipt of T-cell therapy such as that occurring with CAR T-cells, has been found after virus-specific T-cell infusion [20,144]. In fact, Papadopoulou and colleagues reported no elevations in plasma cytokines after multi-virus-specific T-cell infusion [144]. Despite the lack of CRS and infusion toxicity documented, advanced practitioners should maintain vigilant monitoring of any signs of hypersensitivity or infusion-related reaction following virus-specific T-cell infusion.

## 6. Concluding Remarks and Future Perspectives

Viral infections of the CNS are still a major issue in terms of morbidity and mortality among immunocompromised individuals. Antiviral treatments are generally of modest efficacy and strategies reinvigorating antiviral immune activity have been developed, including virus-specific T-cell therapy. Over the recent years, this technique evolved from a theoretic principle to a treatment readily used in some specialized care centers that has shown promising safety and efficacy. Moreover, T-cell therapy also represents a therapeutic hope for patients with virus-associated diseases, such as multiple sclerosis. The key future milestones of virus-specific T-cell therapy probably include standardization of its production and administration, widening of its availability, and improvement of its efficacity.

Concerning treatment standardization, progress has already been made these last few years. Initial case reports and case series are being progressively replaced by Phase I trials aiming at assessing the safety of standardized products. Further trials should assess their optimal regimen, safety, and efficacy.

Third-party virus-specific cell banks for “off-the-shelf” administration hold great promise, making this treatment approach more widely available. However, algorithms for best product selection are not readily established. A higher number of HLA matches between specific T-cells and the recipient correlates with better in vivo proliferation and superior efficacy [146], although even less closely matched products can be effective despite limited persistence after infusion [137]. Studies that aim at determining the optimal T-cell product for each situation will be essential in this respect.

Despite promising results, some patients still fail to respond to virus-specific T-cell administration. The mechanisms underlying this resistance are not fully understood and are still under investigation. Since TGF-beta dampens T-cell response, TGF-beta receptor blockade to render the virus-specific T-cell resistant to the effects of this cytokine, either by gene manipulation or by co-administration of inhibitory molecule, is currently under development [137,147]. Chronic antigen stimulation, for instance in the setting of chronic viral infection, may lead to the development of immune exhaustion, a state of immune cells characterized by specific metabolic and epigenetic status, increased expression of inhibitory checkpoint molecules and reduced activity [148]. Immune checkpoint inhibitors, which are widely being used for the treatment of several cancers and more recently, for the treatment of opportunistic viral infections such as PML, might be used in combination with virus-specific T-cells to reinvigorate antiviral immune activity more efficiently. Finally, many patients who develop opportunistic infections of the CNS are treated with immunosuppressive treatments. In certain clinical situations such as severe GvHD or solid organ transplantation, it might not be possible to withdraw immune suppression, thereby rendering T-cell infusion useless. Gene manipulation to inactivate the glucocorticoid receptor or make cells resistant to calcineurin inhibitors is being developed to maintain activity in the presence of immunosuppressive molecules. Hopefully, this will allow more effective treatment in patients for whom keeping a certain degree of immune suppression is mandatory [149,150,151].

## Figures and Tables

**Table 1:** Patients with progressive multifocal leukoencephalopathy treated using polyomavirus-specific T-cells reported in the literature [34,35,40,41,42,43,44,45,46].

Article	Age (Years), Sex	Underlying Immune Deficiency	Virus Targeted by the Cells	Autologous or Allogeneic Cells	Number of Infusions	HLA Matching	Associated Treatments	Complications	Clinical Outcome Regarding PML
Balduzzi et al., 2011 [34]	14, M	Acute lymphoblastic leukemia	HPyV2	Allogeneic (HSCT donor)	2	10/10	Cidofovir, citalopram	None	Improvement
Muftuoglu et al., 2018 [35]	32, F	Acute myeloblastic leukemia	HPyV1	Allogeneic	3	5/10	Mirtazapine (stopped)	None	Improvement
Muftuoglu et al., 2018 [35]	73, F	Polycythemia rubra	HPyV1	Allogeneic	2	4/10	None	IRIS	Stabilization
Muftuoglu et al., 2018 [35]	35, M	HIV	HPyV1	Allogeneic	5	5/10	ART	None	Improvement
Steinhardt et al., 2020 [44]	59, M	Multiple myeloma	HpyV2	Allogeneic (HSCT donor)	1	10/10	Cidofovir, mirtazapine, DLI	None	Stabilization
Berzero et al., 2021 [41]	59, F	Non-Hodgkin lymphoma	HpyV2	Allogeneic	4	Unknown	None	None	Stabilization
Berzero et al., 2021 [41]	55, M	Multiple myeloma	HpyV2	Autologous	3	Not appropriate	Mirtazapine (stopped)	None	Improvement
Berzero et al., 2021 [41]	70, F	Non-Hodgkin lymphoma	HpyV2	Autologous	1	Not appropriate	None	None	Deterioration and death
Berzero et al., 2021 [41]	50, M	Hodgkin lymphoma	HpyV2	Allogeneic	3	Unknown	Cidofovir (stopped)	None	Unknown (death due to VZV infection)
Berzero et al., 2021 [41]	68, M	Non-Hodgkin lymphoma	HpyV2	Autologous	6	Not appropriate	None	None	Improvement
Berzero et al., 2021 [41]	54, M	Non-Hodgkin lymphoma	HpyV2	Allogeneic	2	Unknown	None	None	Deterioration and death
Berzero et al., 2021 [41]	66, M	Chronic lymphocytic leukemia	HpyV2	Allogeneic	4	Unknown	None	None	Deterioration and death
Berzero et al., 2021 [41]	54, F	Idiopathic CD4 lymphocytopenia	HpyV2	Autologous	6	Not appropriate	None	None	Improvement
Berzero et al., 2021 [41]	17, M	Wiskott–Aldrich syndrome	HpyV2	Allogeneic (HSCT donor)	5	Unknown	None	None	Improvement
Hopfner et al., 2021 [43]	55, F	Hodgkin lymphoma	HPyV1	Allogeneic	4	6/10	None	None	Improvement
Hopfner et al., 2021 [43]	71, F	Breast cancer, dermatomyositis	HPyV1	Allogeneic	3	6/10	None	None	Improvement
Cortese et al., 2021 [40]	62, F	Idiopathic lymphocytopenia, cyclic neutropenia	HPyV1	Allogeneic (two different donors)	3	6/10 (1st) 1/10 (2nd)	Pembrolizumab (stopped), mefloquine	None severe	Deterioration and death
Cortese et al., 2021 [40]	61, F	Microscopic polyangeitis	HPyV1	Allogeneic	3	5/10	Mirtazapine	None severe	Improvement
Cortese et al., 2021 [40]	53, F	Chronic lymphocytic leukemia	HPyV1	Allogeneic	2	10/10	Mirtazapine	None severe	Deterioration and death
Cortese et al., 2021 [40]	71, M	Non-Hodgkin lymphoma	HPyV1	Allogeneic	2	5/10	Cidofovir (stopped), mirtazapine	None severe	Improvement
Cortese et al., 2021 [40]	40, F	Systemic lupus erythrematosus	HPyV1	Allogeneic	3	5/10	None	None severe	Improvement
Cortese et al., 2021 [40]	40, F	Non-Hodgkin lymphoma	HPyV1	Allogeneic	3	6/10	Mirtazapine	None severe	Deterioration and death
Cortese et al., 2021 [40]	60, M	B and D hepatitis	HPyV1	Allogeneic	3	5/10	None	None severe	Stabilization
Cortese et al., 2021 [40]	71, M	Chronic lymphocytic leukemia	HPyV1	Allogeneic	1	2/10	Mirtazapine, mefloquine	None severe	Unknown (withdrew from study)
Cortese et al., 2021 [40]	40, F	Severe combined immunodeficiency	HPyV1	Allogeneic	1	5/10	None	None severe	Deterioration and death
Cortese et al., 2021 [40]	35, F	Common variable immunodeficiency	HPyV1	Allogeneic	2	5/10	Interleukin-7 (stopped), mirtazapine, mefloquine	None severe	Deterioration and death
Cortese et al., 2021 [40]	57, M	Chronic lymphocytic leukemia	HPyV1	Allogeneic	3	10/10	Mirtazapine	None severe	Improvement
Cortese et al., 2021 [40]	72, M	Chronic lymphocytic leukemia	HPyV1	Allogeneic	3	7/10	Mirtazapine	None severe	Stabilization
Wicklein et al., 2021 [42]	57, M	Non-Hodgkin lymphoma	HPyV1	Allogeneic	2	5/10	Pembrolizumab	None	Improvement
Rubinstein et al., 2022 [45]	64, F	Acute myeloblastic leukemia	HPyV1	Allogeneic	6	3/10	None	None	Improvement
Rubinstein et al., 2022 [45]	59, M	Non-Hodgkin lymphoma	HPyV1	Allogeneic (two different donors)	3	2/10 (1st) 3/10 (2nd)	Pembrolizumab (stopped)	None	Deterioration and death
Rubinstein et al., 2022 [45]	62, M	Non-Hodgkin lymphoma	HPyV1	Allogeneic	2	4/10	Unknown	IRIS	Deterioration and death
Rubinstein et al., 2022 [45]	67, F	Non-Hodgkin lymphoma	HPyV1	Allogeneic	1	5/10	Unknown	None	Deterioration and death
Peghin et al., 2022 [46]	29, F	Lung transplantation	HPyV2	Allogeneic	Unknown	5/10	Mirtazapine, mefloquine	None	Improvement

## Data Availability

Not applicable.
